# Supplementary material for: Tead1a Initiates Transcriptional Priming Through the TEAD1a/YAP‐Notch1‐Spi1/Cebpα Axis to Promote Neutrophil Fate
Source: Adv Sci (Weinh). 2025 Aug 28;12(41):e05441. doi: 10.1002/advs.202505441 (PMC12591127; doi:10.1002/advs.202505441)
Supplement: Supplementary file 4 — Supplementary Dataset [file ADVS-12-e05441-s004.zip › 3-6.zip!/3-6/02-6.cluster/cluster17/Enrich/ego_kegg.pdf]

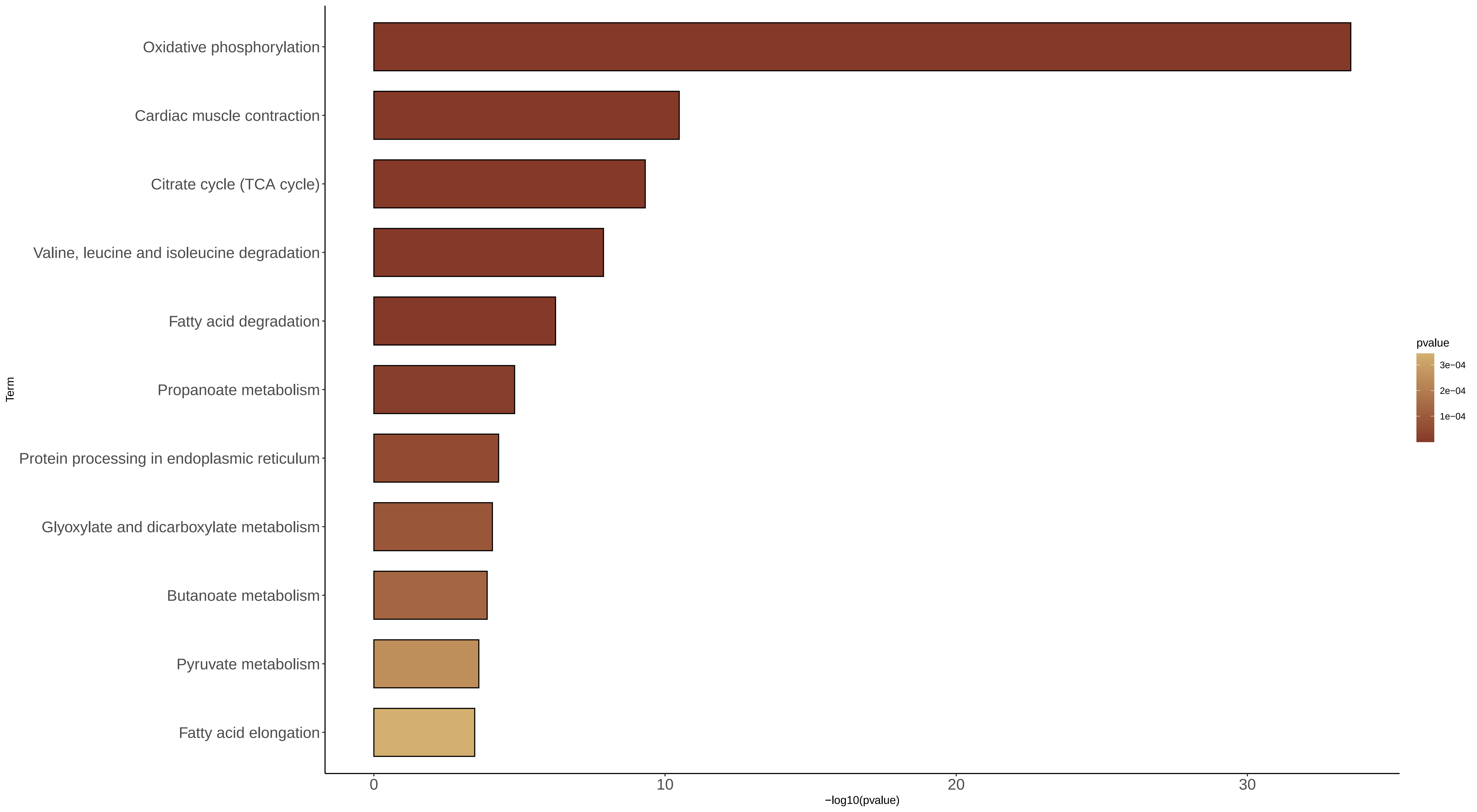
Oxidative phosphorylation
Cardiac muscle contraction
Citrate cycle (TCA cycle)
Valine, leucine and isoleucine degradation
Fatty acid degradation
Propanoate metabolism
Protein processing in endoplasmic reticulum
Glyoxylate and dicarboxylate metabolism
Butanoate metabolism
Pyruvate metabolism
Fatty acid elongation
Term
0
10
20
30
−log10(pvalue)
pvalue
3e−04
2e−04
1e−04
